# Supplementary material for: A Uni-Micelle Approach for the Controlled Synthesis of Monodisperse Gold Nanocrystals
Source: Nanomaterials (Basel). 2024 May 21;14(11):900. doi: 10.3390/nano14110900 (PMC11173505; doi:10.3390/nano14110900)
Supplement: Supplementary file 1 [file nanomaterials-14-00900-s001.zip › nanomaterials-3012407-supplementary.pdf]

Supporting Information

A uni-micelle approach for controlled synthesis of monodisperse gold nanocrystals

Liangang Shan, Wenchao Wang, Lei Qian, Jianguo Tang* and Jixian Liu*

National Center of International Research for Hybrid Materials Technology, College of Materials Science and Engineering, Qingdao University, Qingdao, 266071, P. R. China.

E-mail: ljx@qdu.edu.cn

Supplementary Figureures

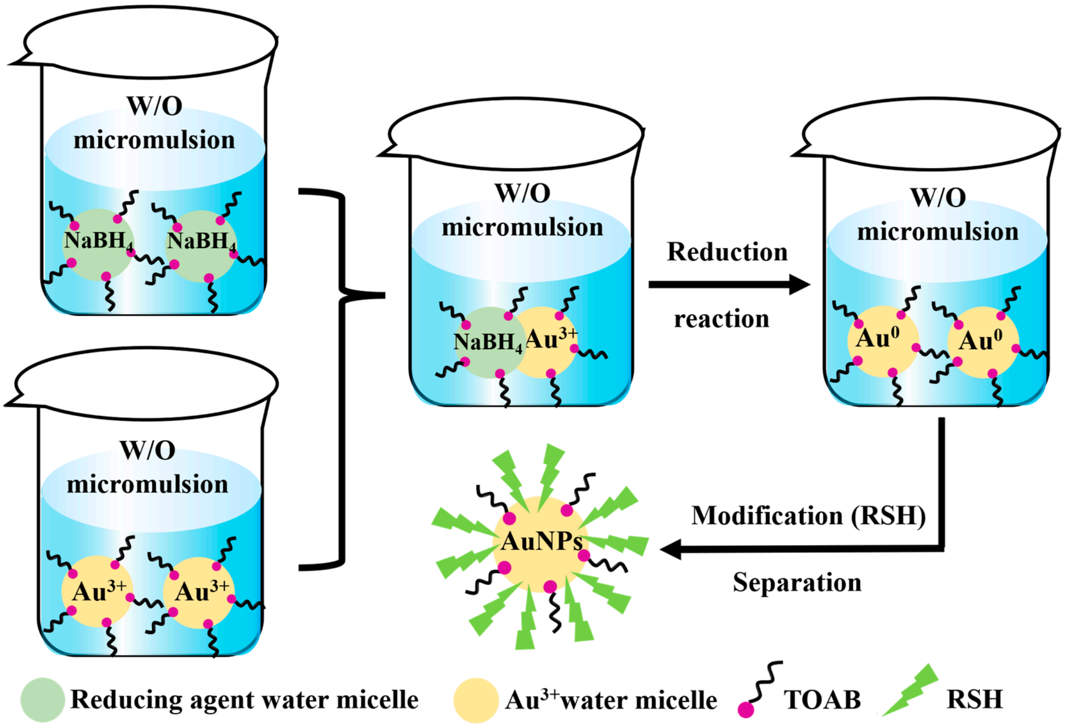

Figure S1. Schematic diagram of AuNPs synthesis via double micelle microemulsion method.

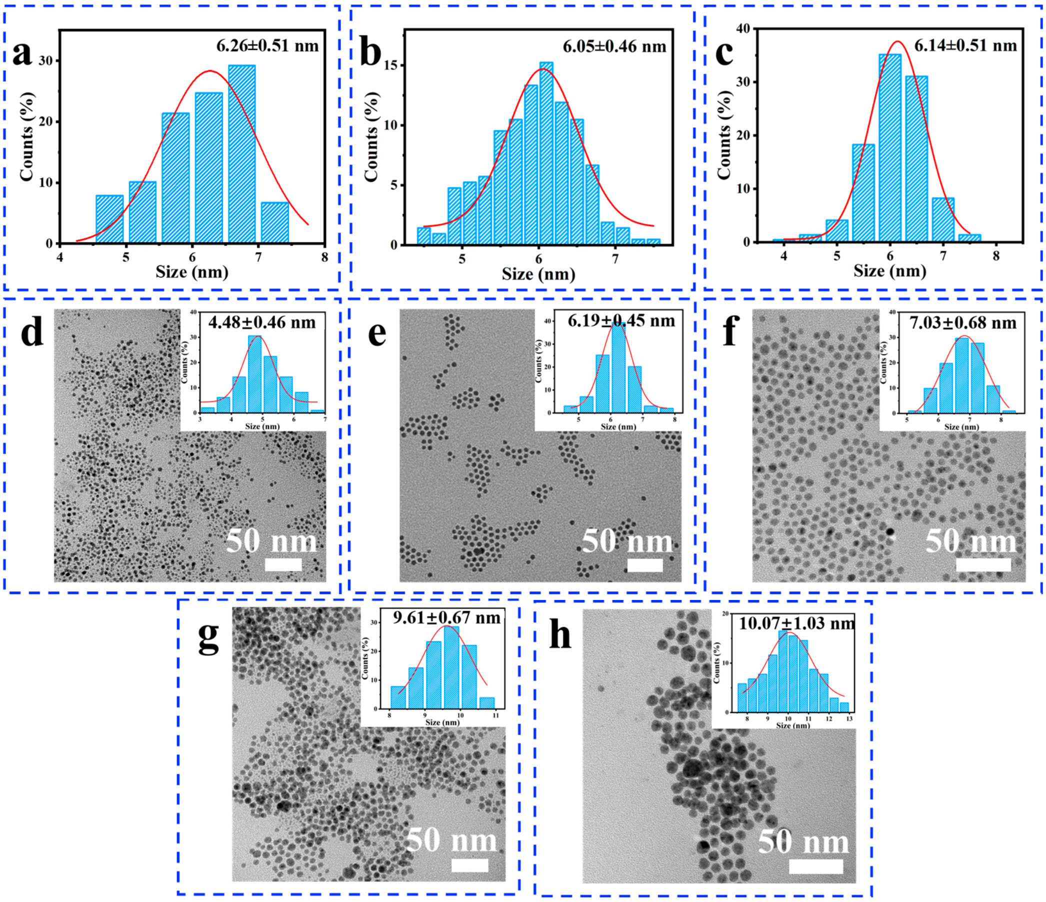

Figure S2. Repeated experiments with water to oil ratio. (a-c) represents three repeated experiments with a fixed value of w = 5, (d-h) represents repeated experiments with w = 2.5, 5, 7.5, 10, 12.5.

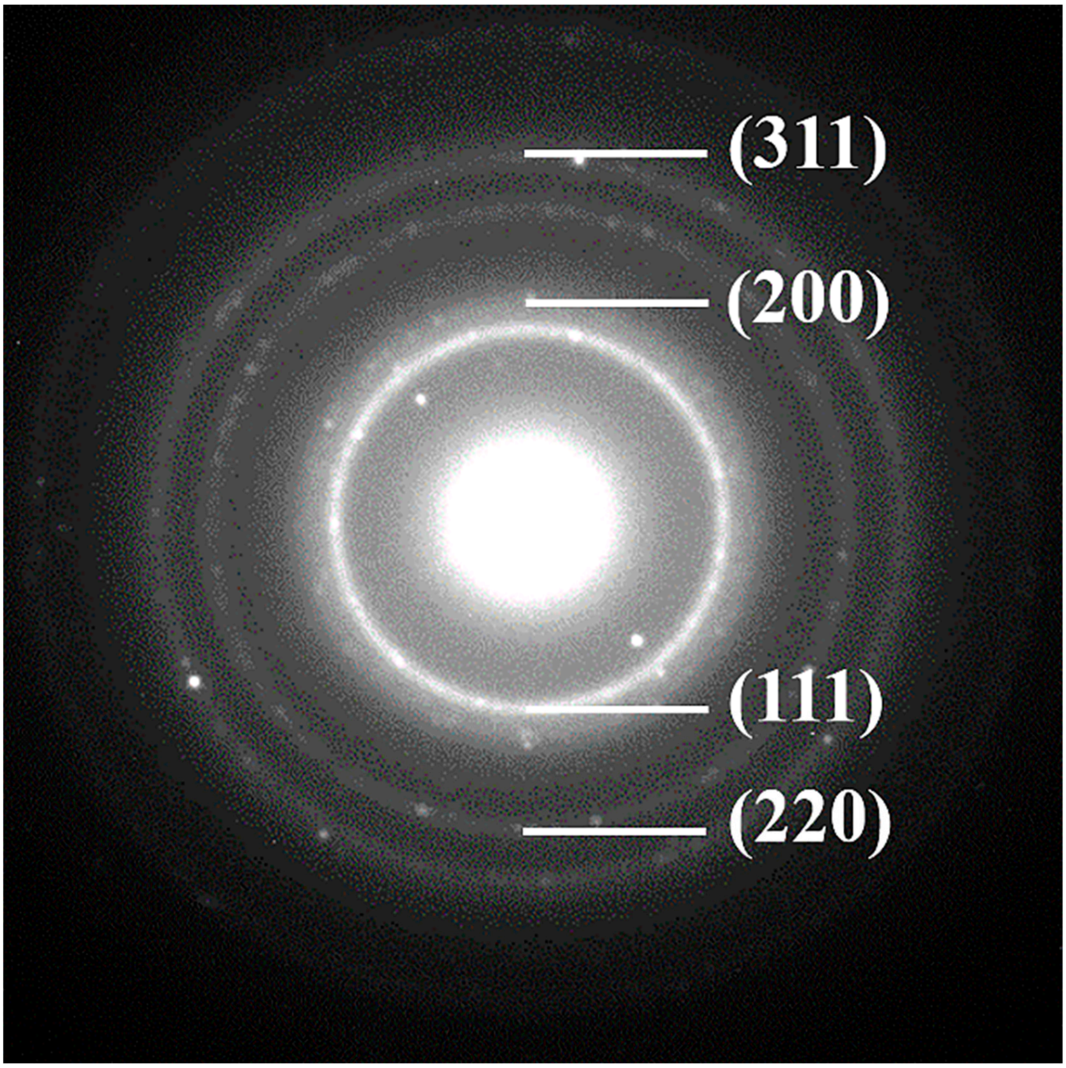

Figure S3. SAED pattern of AuNPs (w = 5).

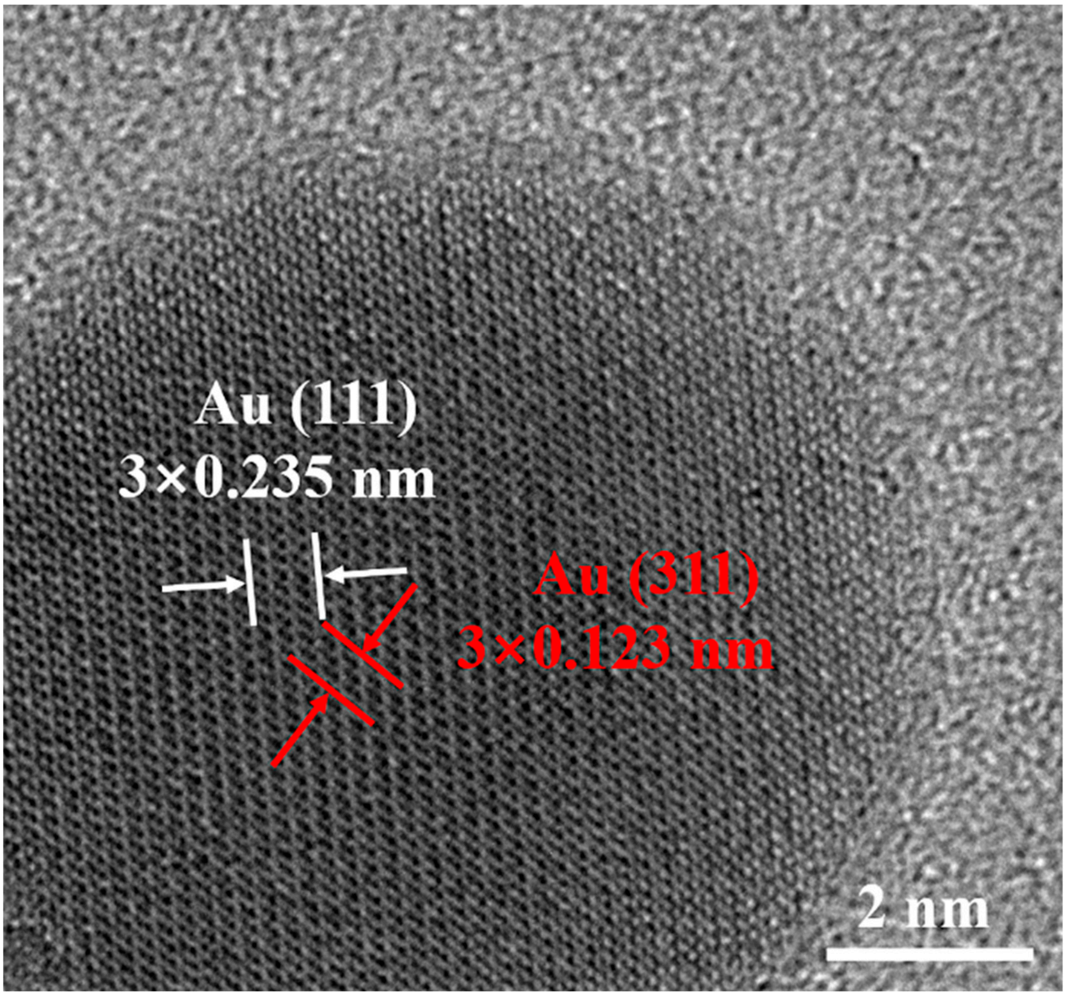

Figure S4. HRTEM images of AuNPs (about 10.54 nm, *w* = 10).

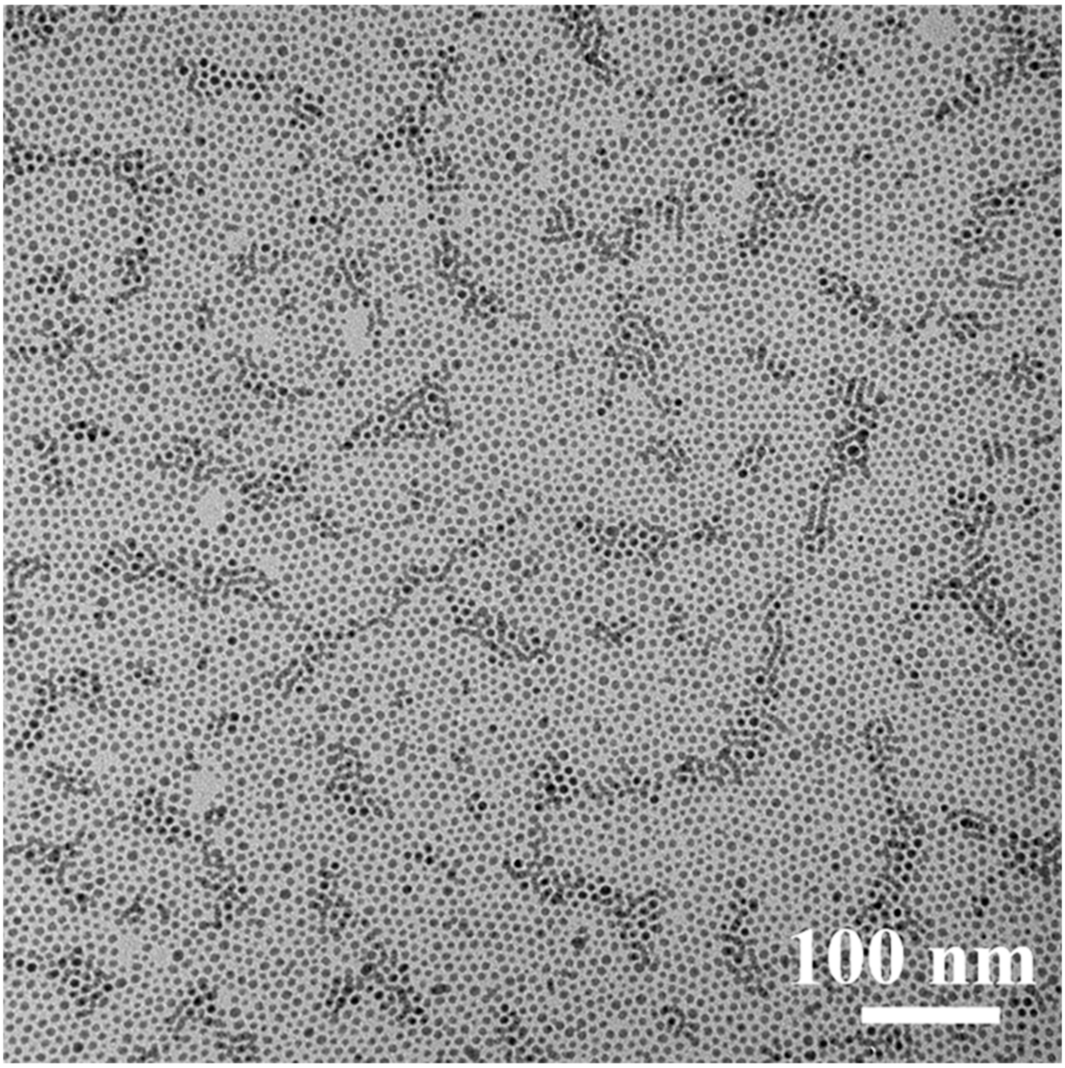

Figure S5. TEM image of AuNPs (about 6.07 nm, *w* = 5).

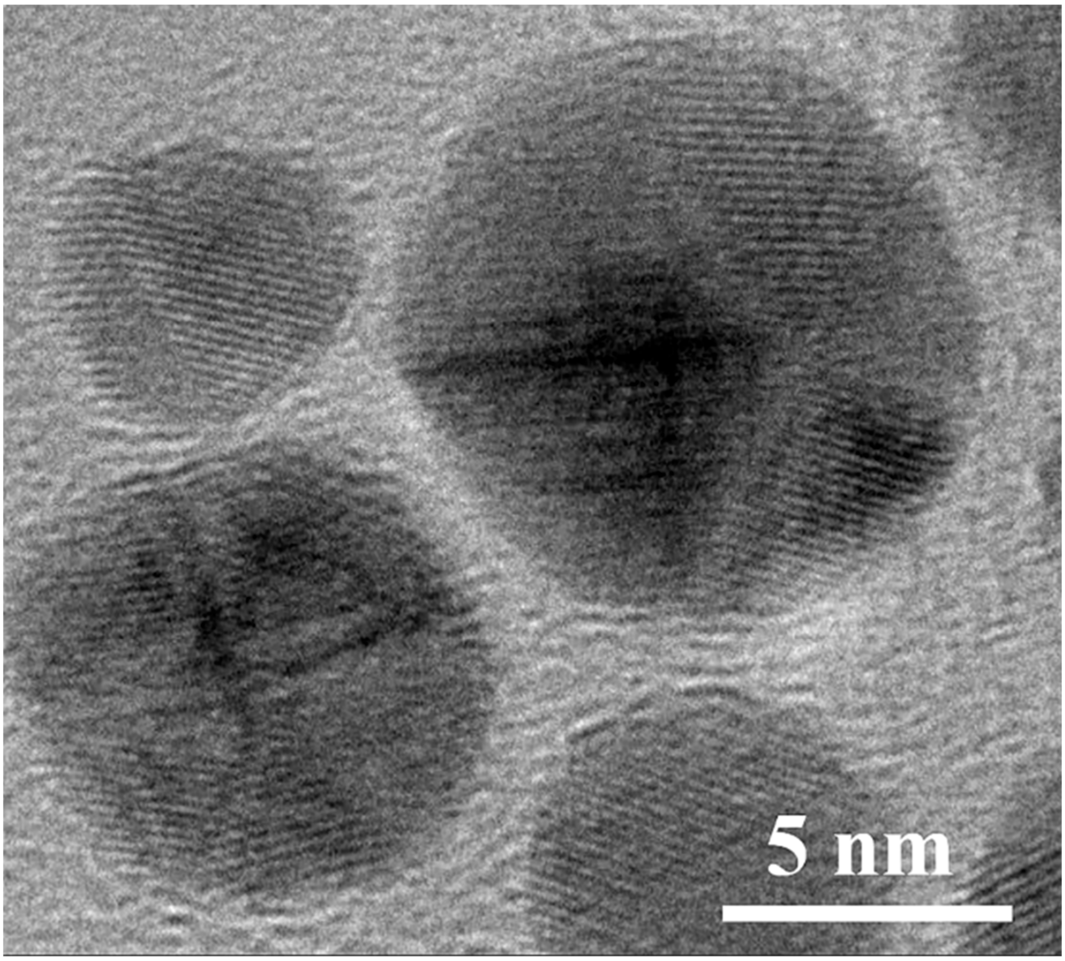

Figure S6. HRTEM image of cyclic penta-twinned gold nanocrystals (w = 5).

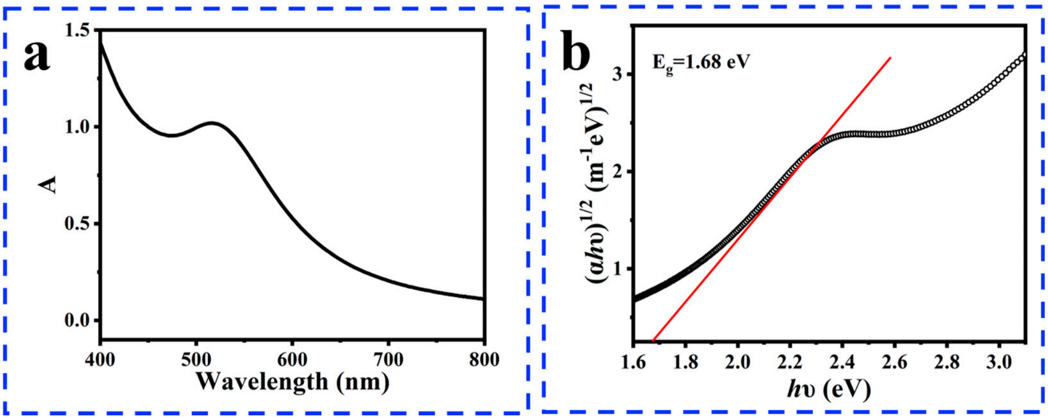

Figure S7. (a) UV spectra and (b) band gap energy calculation from Tauc's plot of AuNPs.

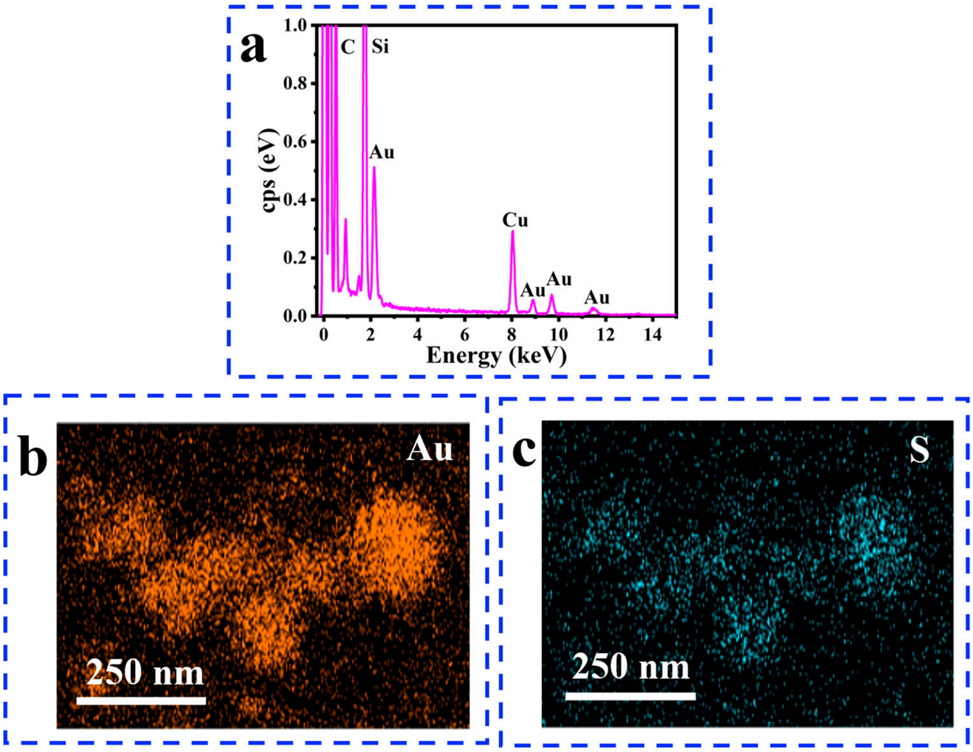

Figure S8. STEM-EDS elemental mappings of AuNPs. (a) The full spectrum, (b) Elemental distribution spectrum of Au, (c) Elemental distribution spectrum of S.

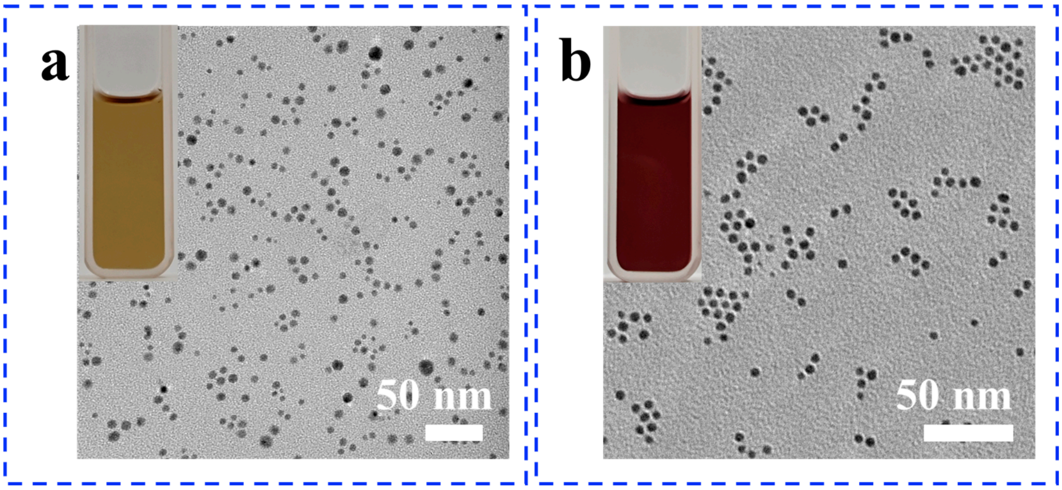

Figure S9. TEM images of AuNPs prepared with different reducing agents, (a) glucose, (b) ascorbic acid. The top left photos are the color of the synthetic solution.

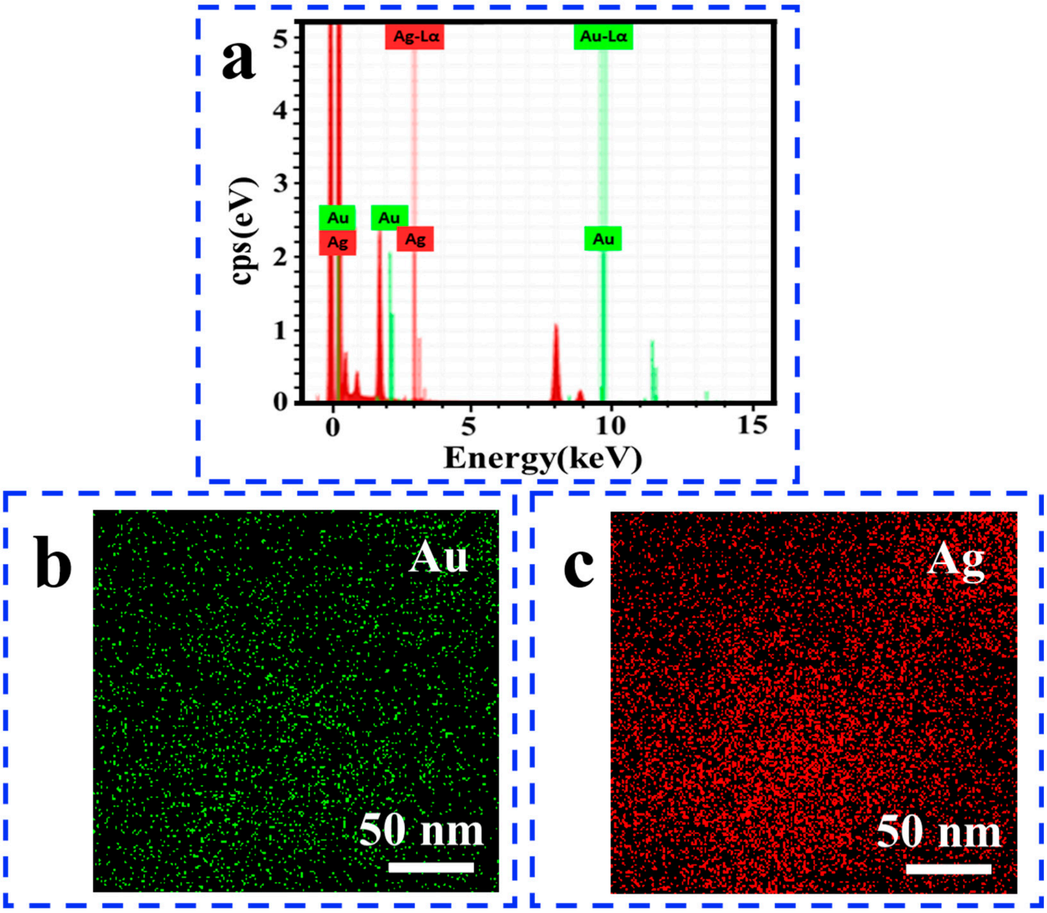

Figure S10. HRTEM-EDS elemental mappings of Au-Ag NPs. (a) The full EDS spectrum, (b) Elemental distribution spectrum of Au, (c) Elemental distribution spectrum of Ag.

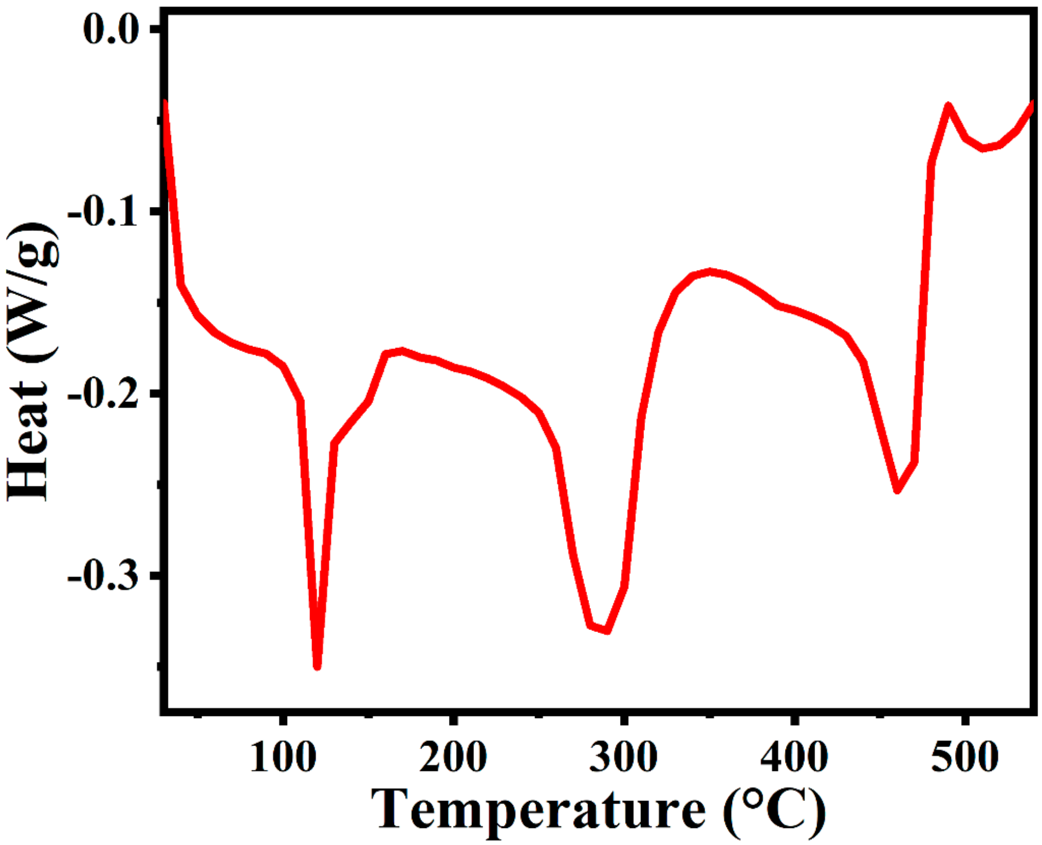

Figure S11. DSC thermograms of AuNPs (6 nm).

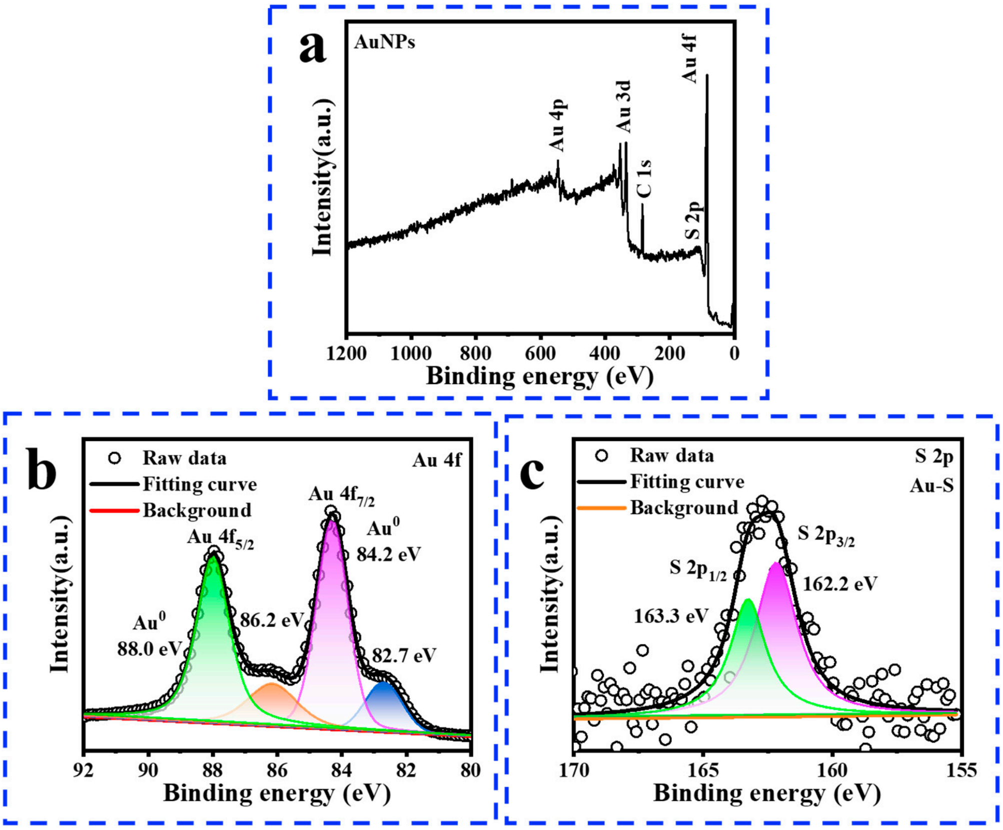

Figure S12. XPS survey spectra of Au(III)-OA. (a) The full spectrum, (b) XPS spectrum of Au, (c) XPS spectrum of N.

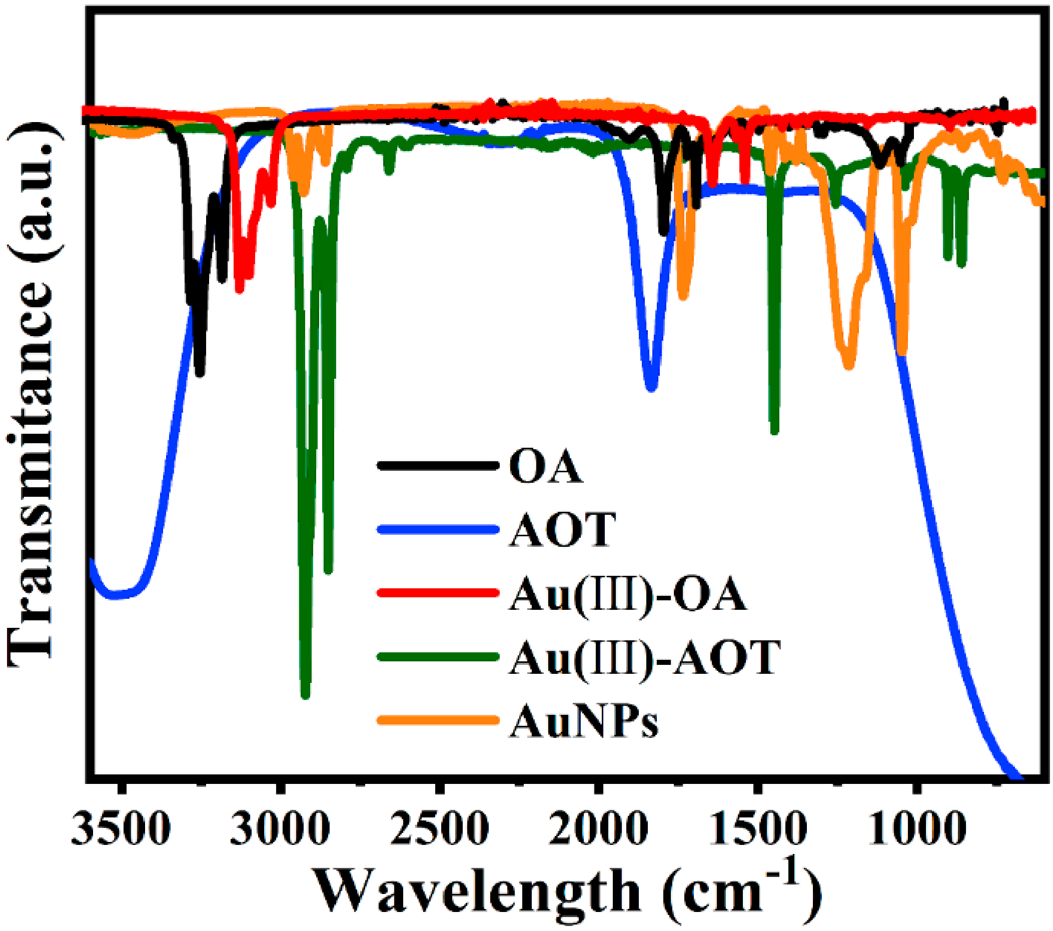

Figure S13. FTIR spectra of products at different reaction stages.

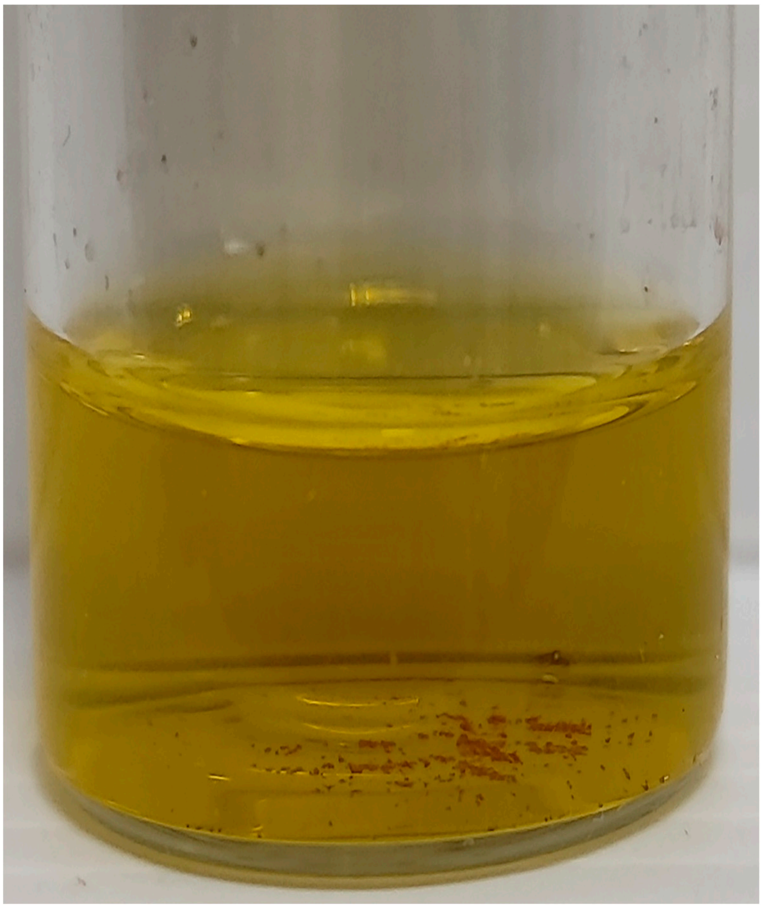

Figure S14. The photo of Au(III)-AOT precipitation.

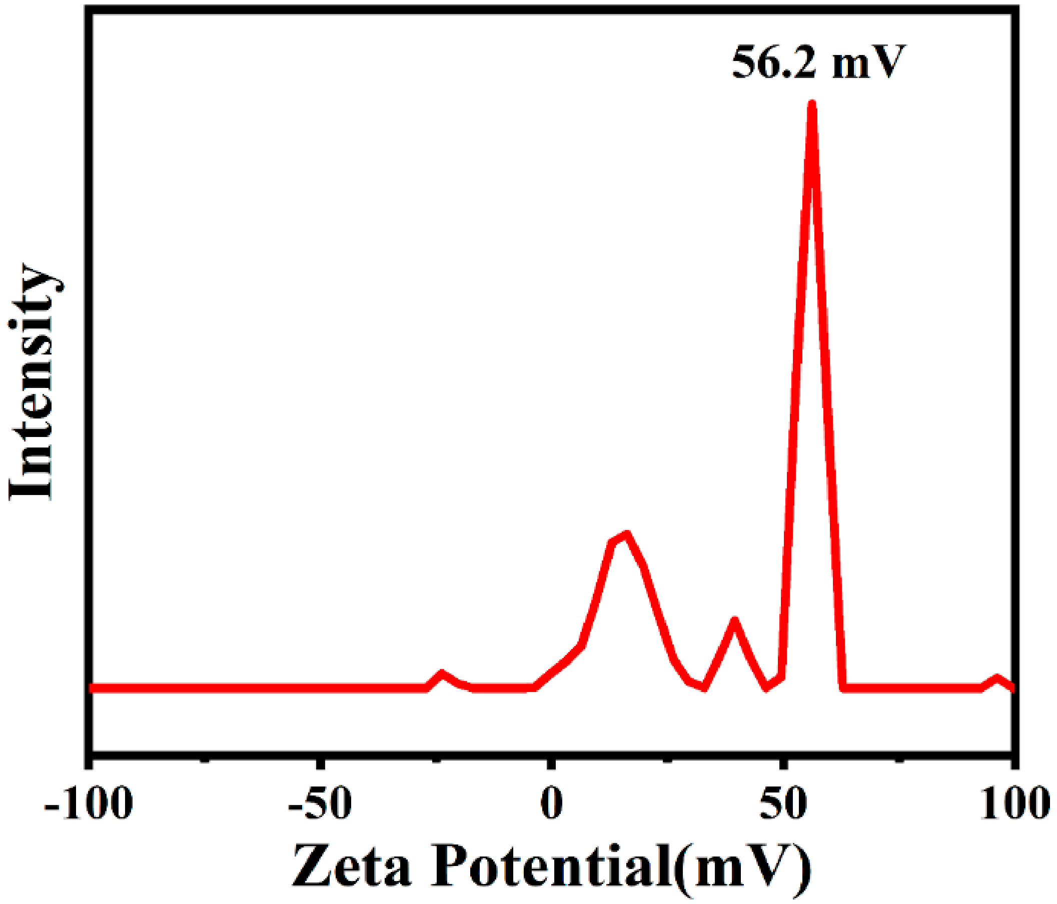

Figure S15. Zeta potential of AuNPs (w = 5).
